# What Is the Role of Assessing Ischemia to Optimize Therapy and Outcomes for Patients with Stable Angina and Non-obstructed Coronary Arteries?

**DOI:** 10.1007/s10557-021-07179-x

**Published:** 2021-05-12

**Authors:** Colin Berry, Andrew J. Morrow, Mario Marzilli, Carl J. Pepine

**Affiliations:** 1 British Heart Foundation Glasgow Cardiovascular Research Centre, University of Glasgow, Glasgow, UK; 2 Golden Jubilee National Hospital, Clydebank, UK; 3 Division of Cardiovascular Medicine, Cardiothoracic Department, Pisa University Medical School, Pisa, Italy; 4 Division of Cardiovascular Medicine, University of Florida, Gainesville, FL USA

**Keywords:** Chest pain, Angina, Coronary heart disease, Ischemic heart disease, Microvascular angina, Vasospastic angina

## Abstract

Ischemic heart disease (IHD) is a leading global cause of ill-health and premature death. Clinical research into IHD is providing new insights into the pathophysiology, epidemiology and treatment of this condition. The major endotypes of IHD include coronary heart disease (CHD) and vasomotor disorders, including microvascular angina and vasospastic angina. Considering unselected patients presenting with stable chest pain, the pre-test probability of CHD is higher in men whereas the pre-test probability of a vasomotor disorder is higher in women. The diagnostic accuracy of diagnostic tests designed to assess coronary anatomy and disease and/or coronary vascular function (functional tests) differ for coronary endotypes. Clinical management should therefore be personalized and take account of sex-related factors. In this review, we consider the definitions of angina and myocardial ischemia. We then appraise the mechanistic links between myocardial ischemia and anginal symptoms and the relative merits of non-invasive and invasive diagnostic tests and related clinical management. Finally, we describe the rationale and importance of stratified medicine of IHD.

## Introduction

Ischemic heart disease (IHD) is a leading global cause of ill-health and premature death [1]. Clinical research into IHD is providing new insights into the pathophysiology, epidemiology and treatment of this condition.

In this review, we consider the definitions of angina and myocardial ischemia. We then appraise pathophysiology, focusing on the mechanistic links between myocardial ischemia and anginal symptoms, the difference between coronary heart disease (CHD) and IHD, and the relative merits of non-invasive diagnostic tests and related clinical management. Finally, we describe the evidence-based treatment of IHD. We consider angina after percutaneous coronary intervention and stratified medicine of IHD.

## Understanding the Definitions of Disease

### Stable Angina

Angina pectoris (derived from the Latin verb ‘angere’ to strangle) is chest discomfort of cardiac origin. William Heberden first characterized the syndrome of angina in 1768; however, it was Allen Burns, who developed the concept that myocardial ischemia causes angina [2].

### Myocardial Ischemia

Myocardial ischemia is a metabolic supply: demand problem [3] that may be transient, recurrent and/or sustained. The causes include inadequate blood oxygen supply due to a general or regional reduction in coronary artery blood flow and impaired myocardial perfusion and reductions in blood oxygen content. Demand ischemia reflects an increase in the metabolic rate secondary to heart rate, body temperature and humoral factors, e.g. catecholamines.

Heusch has argued that myocardial ischemia is a complex phenomenon that is specifically defined by a lack of coronary blood flow (to below 8–10 μl/g per beat) with electric, contractile, metabolic and/or structural consequences for the myocardium, etc [4]. Autoregulation serves to maintain stable coronary blood flow (~80 ml/min/100 g) within the physiological range of blood pressure. Antegrade coronary blood flow mainly occurs during diastole. Under conditions of increased or reduced demand (function, metabolism), the relationship is maintained by the absolute level of blood flow varying according to demand (increased or reduced cardiac function and metabolism). If the perfusion pressure falls below 50 mmHg flow, then autoregulation is depressed and the pressure-flow relationship becomes approximately linear.

## Mechanisms Underlying Angina and Ischemia

### Physiological Regulation of Coronary Blood Flow

Coronary blood flow is regulated by local myogenic, metabolic and endothelial control mechanisms, and the sympathetic nervous system, which induces vasodilation through beta-adrenoceptor activation, and vasoconstriction through alpha-adrenoceptor activation [5]. The endothelium releases vasodilator substances, e.g. nitric oxide and prostacyclin, and endothelial shear stress stimulates production of nitric oxide leading to vascular smooth muscle cell relaxation [6].

### Coronary Artery Disease

Pathological studies led by Drs. Heberden, Burns and colleagues implicated obstructive coronary artery disease (CAD) in the etiology of angina [2]. However, almost two centuries passed until, 1973, K. Lance Gould described the physiological relationship between the extent of coronary artery lumen narrowing and myocardial perfusion [7]. He demonstrated a differential response in coronary artery blood flow at rest versus during hyperemia. A coronary stenosis of 30% could potentially limit hyperemic coronary blood flow whereas resting flow was not affected unless the stenosis was >85%. Califf and colleagues first reported the incremental prognostic significance of the multi-vessel extent and proximal distribution of CAD by stenosis severity (>50% of the lumen diameter) [8].

**Fig. 1:**
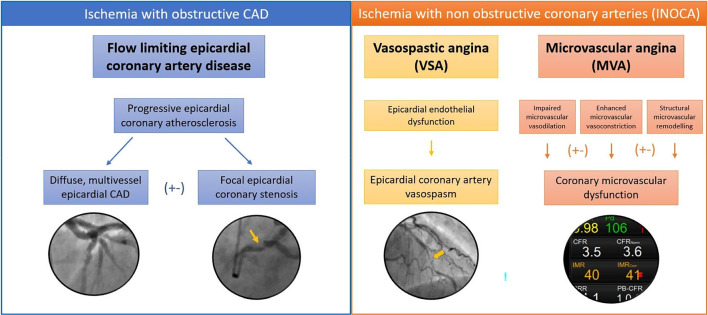
Pathophysiology of myocardial ischemia with or without obstructive coronary arteries

In the presence of diffuse, severe, flow-limiting CAD, physiological stress leads to a coronary blood supply: myocardial demand mismatch and, usually, angina (Fig. 1). This predictable relationship may be altered if a coronary collateral blood supply is well developed, if the myocardium is non-viable, or through effective anti-angina drug therapy.

### Microvascular Dysfunction

The coronary microcirculation includes the resistance arterioles (40–400 μm) and capillaries. Intramyocardial arterioles pass from the epicardial conduit arteries to the sub-endocardium where they form a sub-endocardial plexus, first described by William Fulton [9]. Resistance arterioles provide proportionately the greatest contribution to coronary resistance and are the final common path for myocardial perfusion. The proportion of total resistance in epicardial arteries is negligible; small arteries account for 20% and arterioles are the largest accounting for 40% [5].

Functional and/or structural problems in the microcirculation underlie a susceptibility to myocardial ischemia and microvascular angina [5, 10, 11]. Microvascular dysfunction, reflected by impaired vasodilator capacity, remodeling, rarefaction or extrinsic factors, i.e. fibrosis, leads to impairment of myocardial perfusion, particularly under conditions of physiological stress [12]. Structural microvascular remodeling leads to an increase in microvascular resistance that may cause inducible impairments of myocardial perfusion and symptoms that are like those caused by obstructive CAD. Functional microvascular angina, due to an impaired CFR or microvascular constriction, may give rise to symptoms that are spontaneous and less typical for effort angina.

### Vasomotor Disorders

Vasospasm reflects enhanced coronary reactivity and is due to an imbalance in vasodilator endothelial function and vasoconstrictor vascular smooth muscle cell tone. Sympathetic nervous system tone (either centrally [PVN] or cardiac), activation of the renin angiotensin aldosterone system and endothelin dysregulation are implicated in coronary spasm. Genetic factors , female sex, smoking, inflammation, and illicit drug (cocaine, etc.) associations are also relevant [13–17].

Spasm occurring in a conduit coronary artery and/or its microcirculation will limit myocardial blood flow. Vasospastic angina may occur spontaneously or in association with physiological stress, e.g. exercise, or after eating, following emotional stress, or in association with circadian variations, e.g. during sleep. The symptoms may include chest discomfort, breathlessness, nausea and malaise, all of which may be atypical for angina. 

### Mixed Syndromes

Symptoms due to myocardial ischemia may arise from a combination of vascular disorders, including abnormalities of microvascular tone, resistance and vasospasm [10].

### Coronary Collateral Connections

Collateral connections provide an alternative vascular pathway to myocardium subtended by an obstructed coronary artery. Coronary angiography may reveal collateral connections between arteries within the same branch (ipsi-lateral collaterals) or between the left and right coronary arteries (contra-lateral connections). Arteriogenesis is the remodeling of existing arterioles to develop larger, functional collateral connections (donor vessels) to occluded coronary arteries. Arteriogenesis is mainly driven by hydrostatic effects [18]. Angiogenesis reflects new vessel formation in ischemic tissue mainly stimulated by local angiogenic growth factors such as vascular endothelial growth factor (VEGF).

William Fulton provided histological evidence of collateral connections in adults without heart disease. In post-mortem studies, he described a capillary plexus in the sub-endocardium in the heart of adults who had died for non-cardiac reasons. These nascent connections permit redistribution of blood within the sub-endocardium. The vascular density of this collateral network increases in patients with chronic CAD and left ventricular hypertrophy [9]. Therefore, in patients with chronic coronary heart disease (CHD), compensatory homeostatic mechanisms develop to maintain myocardial perfusion and limit myocardial ischemia. The presence of collaterals might explain why anginal symptoms and/or signs of ischemia may not occur in patients with obstructive CHD. Other reasons include metabolic adaptation, pre-conditioning and even physical adaptations, e.g. reduction in physical activity. A more extensive review is beyond the scope of this article.

### Cardiovascular Conditions and Susceptibility to Myocardial Ischemia

Cardiovascular disease exerts complex effects on these relationships. For example, aortic stenosis reduces stroke volume and the duration of diastole is shorter [19]. Left ventricular hypertrophy increases metabolic demand and stroke volume may also be reduced [20]. When ischemia is persistent or recurrent, metabolic adaptation may occur, a process that is called ‘ischemic conditioning’ [21].

## Myocardial Ischemia and Prognosis

### Myocardial Ischemia in Patients with No Obstructive Coronary Arteries (INOCA)

Coronary vasomotor disorders, including microvascular spasm and coronary artery spasm, cause transient, sustained and recurrent episodes of myocardial ischemia. These vascular disorders can be identified under controlled laboratory testing conditions. The Coronary Vasomotor Disorders International Study (COVADIS) group has established diagnostic criteria for microvascular angina [22] and vasospastic angina [23] and these criteria are now recognized in practice guidelines of international societies [24].

MVA and VSA have adverse prognostic implications and are therapeutic targets. However, disease-modifying therapy is generally lacking. Calcium channel blockers are mechanistically targeted to VSA since beta-blockers may antagonize the β2-adrenoceptor which has a vasodilating effect leading unopposed α-adrenoceptor activation [25].

The nuclear sub-study of the COURAGE trial provided evidence that the extent of myocardial ischemia is prognostically important [26]. This relationship was not observed in the ISCHEMIA trial [27]; however, this trial involved a highly selected population. The participants in this trial were selected based on the presence of moderate-severe ischemia; therefore, the distribution of ischemia was truncated, and not normally distributed. Furthermore, multiple test modalities were used. The angiographic extent of CAD is a well-established determinant of prognosis [28]. Ischemia is complex and may be reproducible in myocardium subtended by a flow-limiting stenosis but it may be more variable if caused by a disorder of coronary vasomotion. This explains why patients with angina due to coronary and/or microvascular spasm may have a normal stress test.

The international CLARIFY registry highlighted the importance of symptoms, showing that angina with or without concomitant ischemia was more predictive of adverse cardiac events compared with silent ischemia alone [29]. Other potential drivers of discordance between angina and ischemia include variations in pain thresholds and cardiac innervation (e.g. diabetic neuropathy) [30].

## Ischemia with No Obstructive Coronary Arteries; Clinical Conundrum or Leading Cause of IHD?

A ‘stenosis-centric’ model places a causal link between obstructive CAD, ischemia and angina. This approach has led many clinicians to routinely believe that angina does not occur without obstructive atherosclerotic CAD. Even in clinical trials, angina is discounted if obstructive CAD is absent [31]. The same is true for regulatory agencies like the FDA, which demand reproducable ECG changes of ischemia before considering an anitanginal agent approvable.  Invasive coronary angiography is the reference test for obstructive epicardial CAD. Computed tomography coronary angiography (CTCA) is increasingly recommended in guidelines as the first-line test for the evaluation of patients with recent-onset anginal chest pain.

The study by Gould et al. in 1974 reported there is not a linear relation between lumen area and myocardial perfusion [7]. In more recent studies, in which stenosis severity was assessed by myocardial fractional flow reserve (FFR) and cross-sectional area was measured by intravascular ultrasound, no linear relation was found between these two parameters, with normal FFRs measured even in severely stenotic vessels [32]. Moreover, in a study where myocardial perfusion was assessed by positron emission tomography (PET) and stenosis severity was estimated by CTCA, some patients with complete coronary occlusionwere found to have normal myocardial perfusion, and many patients with fully patent coronary vessels were found to have severe perfusion abnormalities [33]. These findings challenge the diagnostic role for coronary angiography when used in isolation, be it invasive or non-invasive.

According to the latest update of the ESC Guidelines on Chronic Coronary Syndromes, the clinical pre-test probability for obstructive CAD in patients with typical angina, in the age range 50–59, is as low as 32% in men and 13% in women[24]. This suggests that a majority of patients with typical angina do not have obstructive CAD, which is consistent with the findings from multi-center studies [31].

## Systemic Implications of INOCA

Coronary microvascular dysfunction may also be associated with microvascular dysfunction in other organ systems [16], which probably explains why patients with ischemia with no obstructive coronary arteries (INOCA) may experience impaired exercise tolerance, lethargy and gastrointestinal symptoms. Whether patients with INOCA are at increased risk of dementia due to cerebrovascular small vessel disease is unclear and not established, in part, becasue many such patients aloso hypertension.

## Differential Role of Anatomical and Functional Tests to Guide Clinical Decisions: Coronary Heart Disease vs. Ischemic Heart Disease

The diagnostic assessment of patients with stable angina includes options for non-invasive or invasive tests which may involve anatomical or functional measurements, or a combination of both. Anatomical imaging includes CTCA or invasive coronary angiography. These tests are useful to diagnose and exclude CAD, which mostly affects men. Ischemic heart disease includes major endotypes (1) CAD and (2) vasomotor disorders (ischemia with no obstructive coronary arteries (INOCA). Anatomical tests are not useful to diagnose vasomotor disorders, which mostly affect women. Therefore, anatomical and functional imaging tests provide distinctly different information to inform on the presence of CHD and IHD, respectively.

Anatomical coronary artery imaging does not provide information on myocardial ischemia, even if the artery is occluded. Nuclear myocardial scintigraphy (MPS) is the most specific, non-invasive, metabolic test for assessing myocardial ischemia. Functional tests also include intravenous dobutamine stress echocardiography and treadmill exercise testing. Imaging myocardial perfusion reflects the distribution and kinetics of an intravenous contrast agent, such as a gadolinium chelate for dynamic magnetic resonance imaging (MRI). Cardiac PET provides an absolute measurement of blood flow. Radiotracers include ^15^O-water and rubidium-92 (65% first pass extraction) cardiac PET. ^15^O-water has a 100% first pass extraction fraction and a linear relationship with blood flow whereas rubidium-92 has a 65% first pass extraction and a non-linear relationship with blood flow. Both cardiac PET and MRI, however, have relatively limited spatial resolution and are not use very useful for assessment of endothelial dysfunction. Computational fluid dynamics and machining learning applied to invasive coronary angiography have potiential but are not yet reday for clinical use. 

Non-invasive CTCA represents a major recent advance. CTCA has high sensitivity for coronary atherosclerosis, empowering clinicians to prescribe preventive therapies including anti-platelet and statin medications. CTCA has moderately high specificity for classification of obstructive CAD, potentially leading to a false negative diagnosis. CTCA is increasingly used to inform the decision for invasive management in patients with angina who may benefit from either PCI or CABG.

In PROMISE, anatomical and functional strategies were associated with similar effects on health outcomes [34]. In SCOT-HEART, a CTCA-guided strategy was compared with standard care without any additional testing [31]. In this trial, at 5 years, non-fatal MI was reduced with CTCA-guided management, but there was no effect on all-cause mortality [35]. The CTCA strategy came at a cost of double the number of coronary angiograms (non-invasive and invasive) which raises concern about duplicating tests using ionizing radiation, especially relevant in younger women. In the CE MARC-2 trial, compared with a NICE-guideline risk-based clinical strategy, a strategy of either stress perfusion CMR or MPS reduced the risk of protocol-defined unnecessary angiography (i.e. no obstructive CAD) with favorable effects on health economics [36, 37].

Computational techniques enable estimates of myocardial FFR using computed tomography coronary angiography (CTCA), e.g. FFR-CT, or based on the angiogram procedure, e.g. QFR. Guidewire-based measurements provide pressure-derived FFR and non-hyperemic pressure ratios (NHPR) and these tests are recommended in practice guidelines when there is intermediate coronary artery disease or lack of information from non-invasive tests. FFR and NHPR are pressure-derived ratios. They do not measure myocardial ischemia and, unfortunately, these terms are commonly misrepresented in the clinic.

### Non-invasive Diagnostic Tests: Myocardial Ischemia or Coronary Artery Disease

Historically, diagnostic tests that are used in the clinic are predicated on ischemia being caused by obstructive CAD. Test validation has been determined by association (prediction) of the test findings for the presence of obstructive CAD, usually defined by angiography (50% or 70%) or by flow-limiting CAD [38]. The diagnostic accuracy of non-invasive tests for CAD has been extensively covered elsewhere [39–42].

This raises the challenging question of whether ischemia testing is valid in patients without obstructive CAD? For example, coronary spasm is an important cause of angina but if spasm (epicardial and/or microvascular) is not caused by physiological stress, then functional testing with exercise or pharmacological stimuli may lead to false negative results and sub-optimal management [43]. Although the test result for exclusion of obstructive CAD is a true negative, from the perspective of the patient, it is a false negative outcome.

### The Conundrum of Angina in Patients with No Obstructive CAD: Findings from the SCOT-HEART Trial

In SCOT-HEART, 4146 patients referred for investigation of known or suspected angina were randomly assigned to standard care plus CTCA-guided management (*n* = 2073) or standard care (*n* = 2073) without CTCA and follow-up was continued for a median of 1.7 years [31]. In a pre-specified analysis of Seattle Angina Questionnaire and Short Form 12 scores, compared with standard care alone, adding CTCA was associated with less marked improvements in physical limitation (difference − 1.74 (95% confidence intervals (CI), − 3.34 to − 0.14), *p* = 0.0329), angina frequency (difference − 1.55 (− 2.85 to − 0.25), *p* = 0.0198) and quality of life (difference − 3.48 (− 4.95 to − 2.01), *p* < 0.0001) at 6 months. For patients undergoing CTCA, improvements in symptoms were greatest in those diagnosed with normal coronary arteries or who had their preventative therapy discontinued, and least in those with non-obstructive CAD (*p* < 0.001 for all). This result was unexpected. Whilst the reasons underpinning these between-group differences are not entirely clear, one contributing factor could be the false reassurance given to some patients with INOCA and related discontinuation of angina therapy. This result reflects a narrow, stenosis-centric approach whereby patients who did not have obstructive CAD may have had the diagnosis of angina (due to CHD) overturned. Regardless of the reasons, the result refuted the trial hypothesis that symptoms and quality of life would be improved by a CTCA-guided management strategy and the result somewhat conflicts with the NICE-95 guideline update recommendation for CTCA.

In SCOT-HEART, symptoms and quality of life improved less in the CTCA group vs. standard care group, potentially since angina medication was discontinued by protocol in the patients with no obstructive CAD as defined by CTCA, and many of these patients may have had an underlying diagnosis of microvascular angina [44]. This possibility is now being prospectively assessed in the CorCTCA study [45].

### Population Testing for Obstructive Coronary Artery Disease or Myocardial Ischemia?

In unselected, community-based patients with recent-onset chest pain, only a small minority have obstructive CAD. For example, in the SCOT-HEART trial, in which patients presenting with angina and known or suspected angina underwent CTCA, only 1 in 5 patients had obstructive CAD [31]. However, 9% of the study population already had a history of coronary heart disease at baseline. Most participants with angina had an alternative explanation for their symptoms. This trial did not involve ischemia testing noninvasively or invasively; therefore, the functional significance of the CTCA findings, e.g. FFR, CFR, IMR, for patients who may have had ischemic syndromes attributable to microvascular disease, was uncertain.

### Contemporary Advances in Clinical Practice: Focus on Delineating CAD

The narrow, contemporary focus on imaging CAD is highlighted by the U.K. National Institute of Clinical Excellence (NICE) guideline-95 update (November 2016) which recommended CTCA as the first-line diagnostic test in patients without prior CHD who have known or suspected angina [46]. This proposal reflects the results of the PROMISE [34] and SCOT-HEART [31] trials. Compared with standard diagnostic strategies based on functional testing, the use of CTCA is associated with an increased use of preventive medical therapies and percutaneous coronary intervention that are associated with a reduction in MI by 5 years [47].

This evidence is stimulating a reappraisal of contemporary practice guidelines in North America [48]. However, a default strategy of anatomical imaging has major implications. First, a paradigm shift in practice guidelines may introduce new challenges with access to CTCA, and related socio-demographic issues, as has been observed in the UK [49]. Furthermore, diagnostic tests that do not provide information on myocardial ischemia create a gap in the onward management of symptomatic patients without obstructive CAD, the majority of whom are women who are at risk of being discharged from follow-up. Some of these patients may have microvascular or vasospastic angina and the exclusion of obstructive CAD based on the CTCA findings may lead to false reassurance and potentially inappropriate cessation of angina therapy. Patients with ongoing symptoms, not explained by CTCA, should undergo functional testing to reassess the test accuracy of CTCA and evaluate for myocardial ischemia. Another concern is that exclusion of obstructive CAD may lead to discontinuation of antiatherosclerosis prevention therapies, based on the assumption that such patients do not have atherosclerosis. In fact, when IVUS was performed in a sample of such women in the WISE project, more than 80% had moderate coroanry atherosclerosis that concealed by positive  remodeling (Khuddus, et al J. Interv. Cardiol. 2010;23:511-19). 

## Test for Myocardial Ischemia Linked to Disease Mechanisms: the Case for Stratified Medicine

The approach to assessing a patient with suspected angina should be personalized. Age, sex, clinical history, vascular risk factors, socio-demographic factors and clinical examination should form an initial risk assessment. First-line clinical tests should include measuring the blood pressure and body weight, and a 12-lead electrocardiogram. In addition, consider assessing for anemia, renal dysfunction, glycemic status, lipids, thyroid disease and high sensitivity troponin.

The next steps merit careful consideration. Test options are dependent on factors specific to the healthcare system and should take account of patient preferences. One of the limitations of functional testing is the stressor may not accurately reflect the precipitating factors for symptoms on an individual patient basis. A major problem in clinical cardiology relates to the scenario of a patient with anginal symptoms, myocardial ischemia on non-invasive testing and no obstructive coronary arteries as revealed by invasive coronary angiography, including if FFR or NHPR rule-out flow-limiting CAD. Many cardiologists would then call into question the specificity of the non-invasive test, query test artefact or doubt the veracity of the patient’s symptoms. The gap in test information relates to the possibility of a disorder of coronary vasomotion, a missed diagnosis of microvascular angina or vasospastic angina, false reassurance and sub-optimal management. Based on many studies, such as CorMicA [10], this scenario appears to be all too common and is promulgated in contemporary practice guidelines [46, 50].

**Fig. 2:**
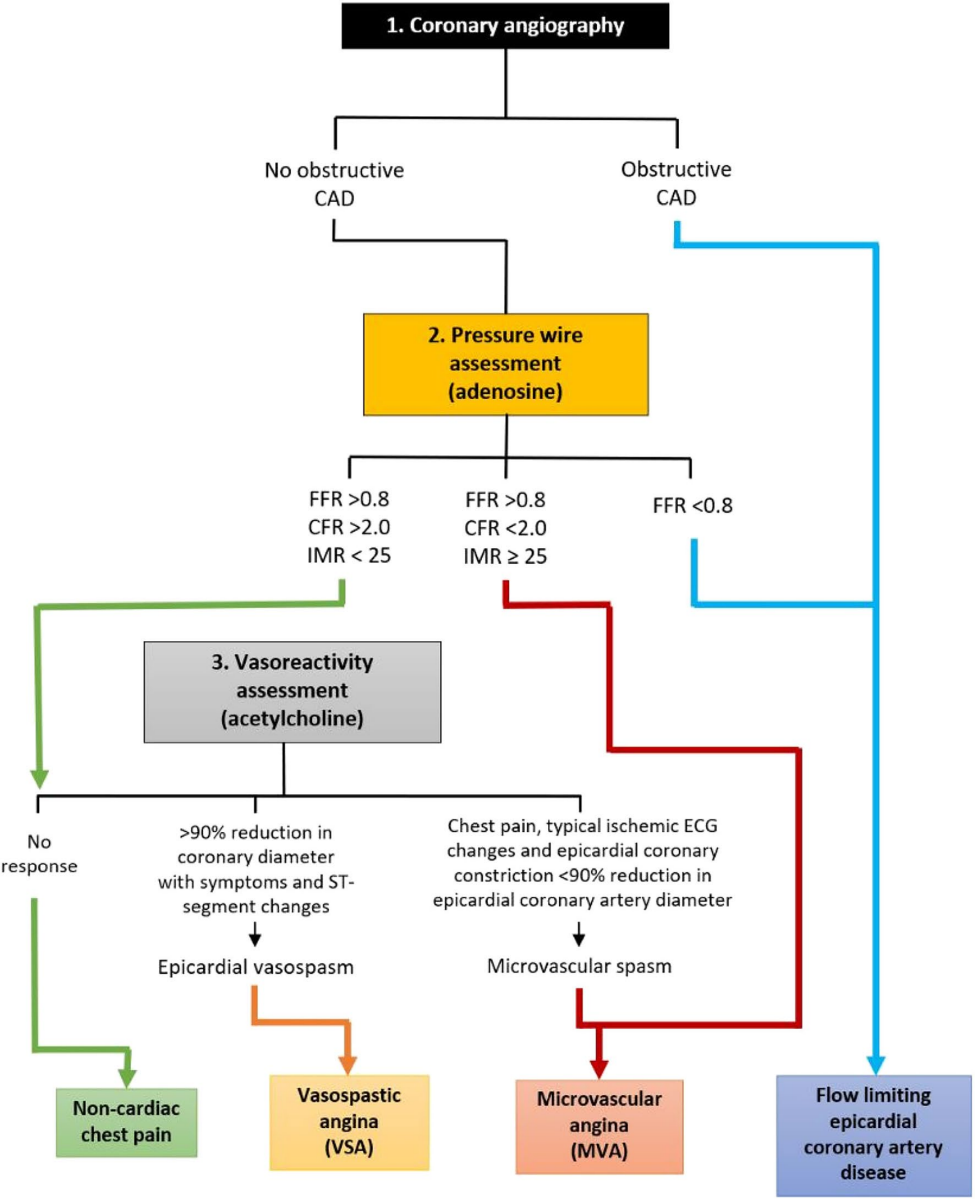
Functional coronary angiography

New practice guidelines highlight the need for clinicians to focus on disease mechanisms [24, 51] (Fig. 2). The clinician should be clear of the question and the strengths and limitations of the diagnostic tests. Of note, dobutamine stress testing is evidence-based for detection of obstructive CAD whereas invasive pharmacological tests using ergonovine and acetylcholine rule-in/rule-out coronary vasomotor problems and endothelial dysfunction [52, 53].

Gaps in clinical practice include the lack of a blood test that might provide specific or incremental information about the presence of INOCA and endotypes. Proteomics and metabolomics hold future promise. The gap relevant to non-invasive anatomical CTCA is the lack of information about coronary vascular function. On the other hand, a gap for non-invasive functional testing is the lack of information about coronary atherosclerosis. FFR-CT bridges this gap but not for small vessel disease.

The list of possible mechanisms for myocardial ischemia goes beyond stenosis, vasospasm and microvascular dysfunction. Pepine and Douglas propose a classification for chronic coronary syndromes broken down into vascular and non-vascular causes [54]. They acknowledge that despite recent advances, gaps remain in our understanding of the mechanisms underlying INOCA and that for many of the possible mechanisms, we may not yet have identified appropriate triggers to replicate in functional testing.

## Evidence-Based Treatment for Ischemic Heart Disease

During the past 50 years, evidence-based treatment of CAD has evolved progressively and aspirin, statins, angiotensin-converting enzyme inhibitors and coronary artery bypass surgery confer survival benefits in indicated patients [55–58]. In patients with multi-vessel and/or left main disease, PCI confers comparable prognostic benefits as compared with CABG. [59, 60]

**Fig. 3:**
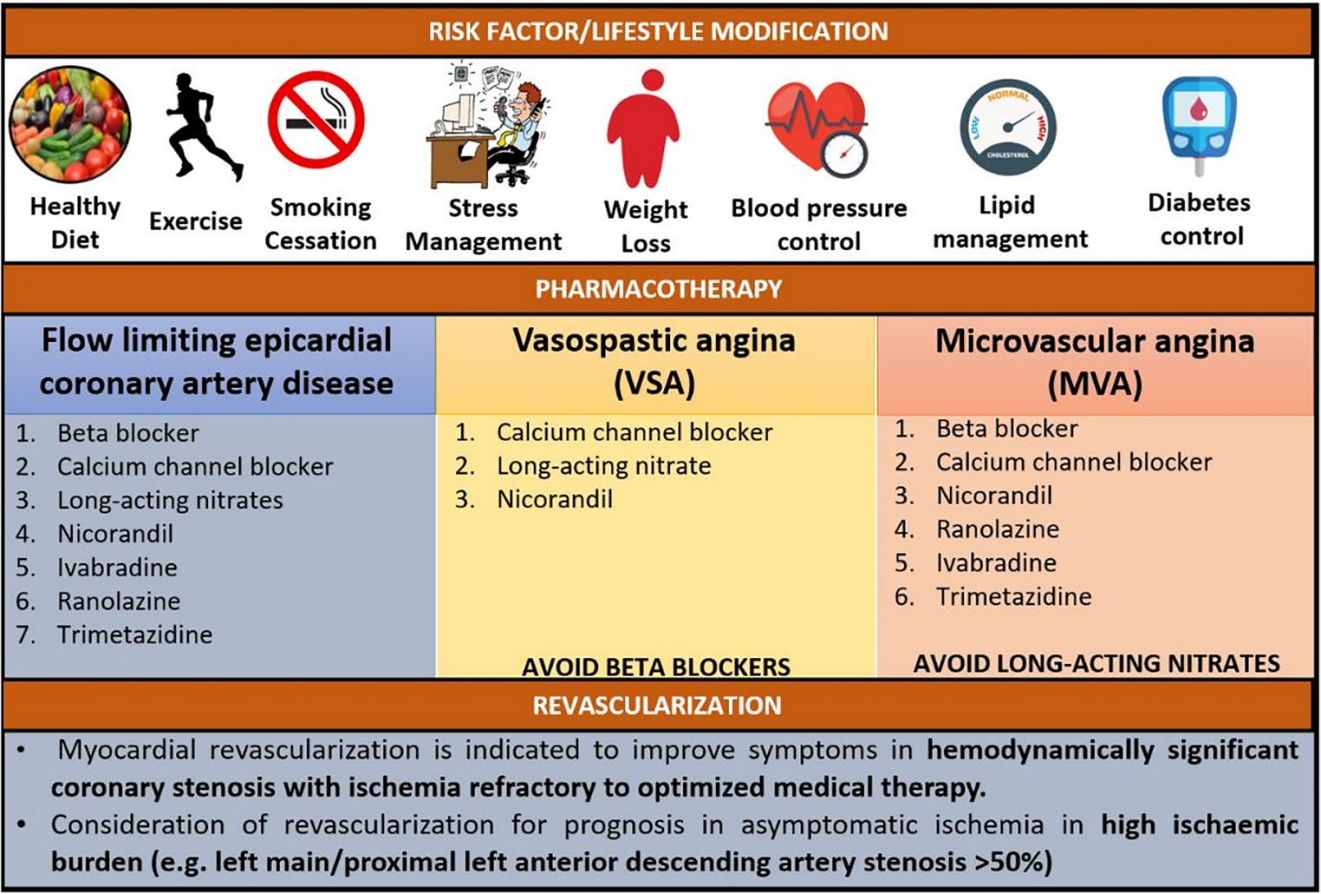
Management of ischemic heart disease

Contemporary medical therapy for angina is mainly based on medicines that were developed in patients with obstructive CAD, notably calcium channel blockers and beta-blockers [24] (Fig. 3). In recent years, relatively few comparative trials of anti-anginal drugs have been undertaken [61]. Nicorandil is a mitochondrial ATP-sensitive potassium channel activator that may be helpful for patients with angina, but supporting evidence is not well-established effects [62–64]. Ranolazine is an inhibitor of late inward sodium current thereby inhibiting calcium overload in ischemic myocardium. Ranolazine add-on therapy may improve exercise capacity and anginal symptoms [65]. Trimetazidine inhibits fatty acid oxidation leading to improvements in myocardial glucose utilization, thereby limiting ischemia. However, recent clinical outcome trials involving novel anti-ischemic therapies in patients with CAD, including ranolazine and trimetazidine, have not translated into convincing clinical benefits [64, 66]. Microvascular angina is a key focus for disease-modifying therapy development. The Precision Medicines in Microvascular Angina (PRIZE) trial will assess whether repurposing zibotentan, an endothelin A receptor-selective antagonist, in population of patients enriched with the G-allele of single nucleotide polymorphism (SNP) rs9349379 endothelin 1 gene enhancer, might improve exercise duration, symptoms and quality of life [67]. PRIZE sets the scene for a pipeline of novel therapies targeted to microvascular disease.

Whilst the evidence for clinical benefits from novel angina drugs is not compelling, clinical strategies involving stepped approaches to optimization of medical therapy have proved efficacious. The COURAGE [68] trial was the first to provide evidence in favor of medical therapy vs. PCI. More recently, the ISCHEMIA [27] and ORBITA [69, 70] trials have provided much more convincing evidence that tailored medical therapy strategies without may initially be sufficient, leaving invasive management as an option for patients with symptoms not controlled by medical therapy.

## Post-PCI Angina

Anatomical and functional causes of ischemic symptoms may co-exist. The presence of a coronary artery stenosis does not prove its causative role [71], although an FFR ≤ 0.80 identifies flow-limiting CAD. In patients with obstructive CAD, microvascular disease may contribute to myocardial ischemia post-PCI (Type 4 Microvascular Angina), which is one reason why angina may persist post-PCI. On the other hand, the DEFINE-FLOW trial (ClinicalTrials.gov Identifier: NCT02328820), recently reported at TCT Connect 2020, served evidence that in patients with flow-limiting CAD (FFR ≤ 0.80) who had a preserved coronary flow reserve, deferral of PCI does not protect against future ischemic events.

A series of landmark clinical trials including COURAGE [68], ORBITA [69] and ISCHEMIA [27] demonstrated that routine invasive management and linked revascularization may not improve prognosis or even relieve angina. The evidence from these trials reflects the actual reports of individual patients in whom angina may persist after technically successful PCI. Several factors may contribute to persistence of angina post-revascularization including residual, untreated CAD, persistence of flow-limiting CAD in the treated artery and microvascular disease (Types 3 and 4 microvascular angina), according to the classification of Camici and Crea [71, 72]. The latter is, arguably, an under-recognized problem, not least since diagnostic techniques to measure microvascular dysfunction have not been widely adopted.

Sometimes, angina medication may not help the patient. This may be explained by a lack of pharmacological effect on the underlying disease mechanism. For example, a beta-blocker may not be helpful in vasospastic angina. It is for this reason that a disease-targeted approach to therapy is needed.

## Stratified Medicine

Clinicians should pursue a diagnostic test strategy that aligns to the patient’s medical history. They should clarify the symptoms, identify the mechanisms and link the treatment with the underlying mechanisms. This approach is called ‘stratified medicine’. If the mechanism is unknown then ischemia testing becomes problematic, especially if the test is negative. If ischemia is evident then knowledge of the mechanism will help inform/stratify therapy. The CorMicA trial was the first to test stratified medicine in angina and the results indicate stratified medicine holds promise for the development of novel therapies in clinical trials [10]. This is because the outcome of the trial, such as an improvement in angina or exercise capacity, can be directly linked to the underlying mechanism. For example, the Precision Medicine With Zibotentan in Microvascular Angina is a randomized, placebo-controlled, clinical trial of treatment for 12 weeks with the endothelin A receptor-selective antagonist, zibotentan [67]. The eligibility criteria require evidence of myocardial ischemia and/or microvascular dysfunction according to the COVADIS criteria and in addition, the population is enhanced with the G-risk allele rs9349379, which is a distal regulator of the endothelin gene [13]. The primary outcome is exercise duration on the Bruce treadmill exercise test protocol. Therefore, should anginal symptoms and exercise time improve with zibotentan, then a plausible mechanism would be improvement in microvascular perfusion and relief of myocardial ischemia mediated by endothelin A receptor blockade. Improvement in CFR and angina (SAQ) was documented in a randomized, double blined, trial of high dose ACE inhibition (quinapril, 80 mg/day) versus placebo in a WISE ancillary study (Pauly D, et. al. Am Heart J. 2011;162;678-84). This strategy is being tested in the WARRIOR (**W**omens’ Ischemi**A** T**R**eatment **R**educes Events **I**n Non-**O**bstr**R**uctive CAD) trial (NCT#03417388) randomizing clinically stable women symptoms of ischemia and nonobstructive CAD by invasive coronary angiogram or CTCA to either Intensive Medical Treatment (IMT, centered on high dose ACE-I/ARB) or Usual Care (UC). The primary outcome is death, MI, stroke or hospitalization for angina or heart failure and secondary outcomes include the SAQ and health resouce consumption. (Handberg E, et al. Am Heart J. 2021;237:90-103). 

## Conclusions

Most patients presenting with angina do not have obstructive CAD. If anatomical imaging with CTCA or invasive angiography is undertaken first, patients with INOCA should be considered for functional testing. This should clarify the underlying disease mechanisms enabling linked treatment decisions for personalized treatment decisions (stratified medicine).

## Data Availability

Not relevant. There are no research data related to this review article.
